# We will not end AIDS: addressing the anti‐rights movements

**DOI:** 10.1002/jia2.26429

**Published:** 2025-02-28

**Authors:** Allan Maleche, Wame Jallow, Jerop Limo, Timothy Wafula, Solomon Wambua

**Affiliations:** ^1^ Kenya Legal and Ethical Issues Network on HIV & AIDS (KELIN), Gender and Rights Advisory Panel (GAP) & UNAIDS Reference Group on HIV and Human Rights Nairobi Kenya; ^2^ Independent Health Activist Gaborone Botswana; ^3^ Ambassador for Youth and Adolescent Rep Health Programme (AYARHEP) Nairobi Kenya; ^4^ Kenya Legal and Ethical Issues Network on HIV & AIDS (KELIN) Nairobi Kenya; ^5^ Key Population Consortium Nairobi Kenya

1

In 2015, global leaders made an ambitious commitment to end the AIDS epidemic under the 2030 Agenda for Sustainable Development, through political will, investments and rights‐based approaches [1]. On Zero Discrimination Day 2025, we sound the alarm on the growing wave of anti‐rights and anti‐gender movements that now threaten to roll back of these gains, putting millions of lives at risk. Anti‐gender movements refer to organized efforts aimed at opposing gender equality and the rights of marginalized groups, particularly those advocating for sexual and reproductive health rights and the rights of LGBTIQ+ communities. These movements often target policies and programmes that promote gender inclusivity, comprehensive sexuality education and equal access to healthcare, using narratives that reject evolving gender norms and human rights frameworks.

The UNAIDS 2024 report emphasized that the global momentum in ending AIDS hinges on sustained political and financial investments [2]. It highlighted the need to protect human rights, warning that any backtracking will undermine gains in the HIV response. This echoes the Global Commission on HIV and the Law (2012) [3] and the UN Secretary‐General's report (2016) [4], which both reaffirmed that access to HIV services must be ensured for marginalized populations, including people living with HIV, young women in sub‐Saharan Africa, sex workers, men who have sex with men (MSM), transgender people and people who inject drugs.

However, despite it being an established fact that rights‐based strategies are important in ending HIV, 2024 witnessed merciless backlash on those rights. Conservative governments around the world are increasingly posing a threat to human rights, with suppression of human rights defenders, and universal human rights principles and laws being attacked and undermined by these governments.

Most troubling of these trends is the increased criminalization and exclusion of LGBTIQ+ people from healthcare services. In Kenya, for instance, a 2023 Supreme Court decision enabled the registration of the National Gay and Lesbian Human Rights Commission—a landmark victory for human rights [5].

But instead of promoting progress, political and religious leaders utilized the ruling to fuel public outrage, which led to a rise in violence towards the LGBTIQ+ community, closures of health service organizations (mainly drop‐in centres led by MSM) and interruption of outreach initiatives to key population communities.

Uganda took it a step further. The passing of the Anti‐Homosexuality Act in 2024 effectively criminalized LGBTIQ+ livelihoods, with disastrous consequences for HIV prevention and treatment. East African civil society groups warned that such laws push people underground, where they cannot access basic health services [6]. Ghana followed the same route with a similar bill, which was not signed into law. Kenya also made legislative attempts to further criminalize and discriminate against the LGBTIQ+ community.

These laws not only legitimized discrimination, fuelled stigma and pushed entire communities away from HIV services; they increased their vulnerability to violence, discouraged HIV testing and blocked access to treatment—effectively making the goal of ending AIDS by 2030 far less attainable.

Apart from attacks on LGBTIQ+ rights, anti‐rights movements are also rolling back reproductive health policies, further threatening progress on HIV prevention. Kenya's National Reproductive Health Policy (2022−2032) is an example of the implications of this rollback [6]. The policy excludes unmarried women and young people from accessing reproductive health information, raising the legal age for accessing some of the services from 21 to 25 years. The ban disproportionately affects young women, who already bear the brunt of incident HIV acquisitions in sub‐Saharan Africa. Moreover, the Kenya Obstetrics and Gynaecological Society has raised concerns that requiring parental consent for adolescents discourages youth from seeking care, exposing them to higher risks of HIV, unwanted pregnancy and gender‐based violence [7].

The re‐imposition of the Global Gag Rule in 2025 [8]—a policy withholding U.S. funds from foreign organizations that provide or even discuss abortion services—is another major setback. The policy weakens reproductive health systems, as organizations that receive funding from the U.S. government will find it harder to offer comprehensive health services, particularly those focusing on access to safe abortion. The impact will be especially catastrophic in low‐income nations, where many health systems rely on international funding to sustain essential health infrastructure.

Paradoxically, even while Kenya's government launched the Triple Threat Campaign [9]—to address new HIV acquisitions, teen pregnancies and gender‐based violence—it has yet to answer how restrictive policies like the Reproductive Health Policy are fuelling exactly these same issues. The lack of any comprehensive sexuality education in Kenyan schools only exacerbates the problem, and teens have neither the knowledge nor the tools to defend themselves.

Apart from LGBTIQ+ discrimination and reproductive rights, a pressing issue is the continued criminalization of HIV transmission, exposure and non‐disclosure. Despite overwhelming evidence showing that such laws do not consider actual risks of transmission—especially with the effectiveness of antiretroviral therapy—many countries still prosecute individuals even when no harm was inflicted.

These laws not only fuel stigma but also deter individuals from being tested—for fear that if they test positive for HIV, they could be open to prosecution. The result? More people will not know that they are living with HIV, more people will be untreated and more people will acquire HIV.

This Zero Discrimination Day, we must confront the brutal fact: anti‐rights and anti‐gender movements are directly threatening the HIV and health response, more so now with changes in the U.S. government leadership. This will increase stigma, fuel discrimination and restrict access to essential services—reversing decades of progress.

To reverse the trend and stay on track to end AIDS by 2030, we need:

· **Governments** to repeal discriminatory laws that criminalize HIV status, restrict reproductive rights and prevent LGBTIQ+ individuals from accessing healthcare.
· **Stronger political leadership** to protect human rights and sustainably fund HIV responses.
· **Donors** to prioritize funding community‐led advocacy that responds to anti‐rights policies and strengthens service delivery.
· **A reinvestment in human rights‐based strategies**, ensuring health equity, access to services and legal protections for vulnerable groups.

Ending AIDS is not just a scientific goal—it is a question of justice. Without upholding human rights, the vision of an AIDS‐free world by 2030 will be nothing but an empty promise.

## COMPETING INTERESTS

The authors declare no competing interests.

## AUTHORS’ CONTRIBUTIONS

AM, WJ, JL, TW and SW contributed to the analysis, writing and review of the article.
